# Supplementary material for: Excess extracellular K+ causes inner hair cell ribbon synapse degeneration
Source: Commun Biol. 2021 Jan 4;4:24. doi: 10.1038/s42003-020-01532-w (PMC7782724; doi:10.1038/s42003-020-01532-w)
Supplement: Supplementary file 1 — Supplementary Information [file 42003_2020_1532_MOESM1_ESM.pdf]

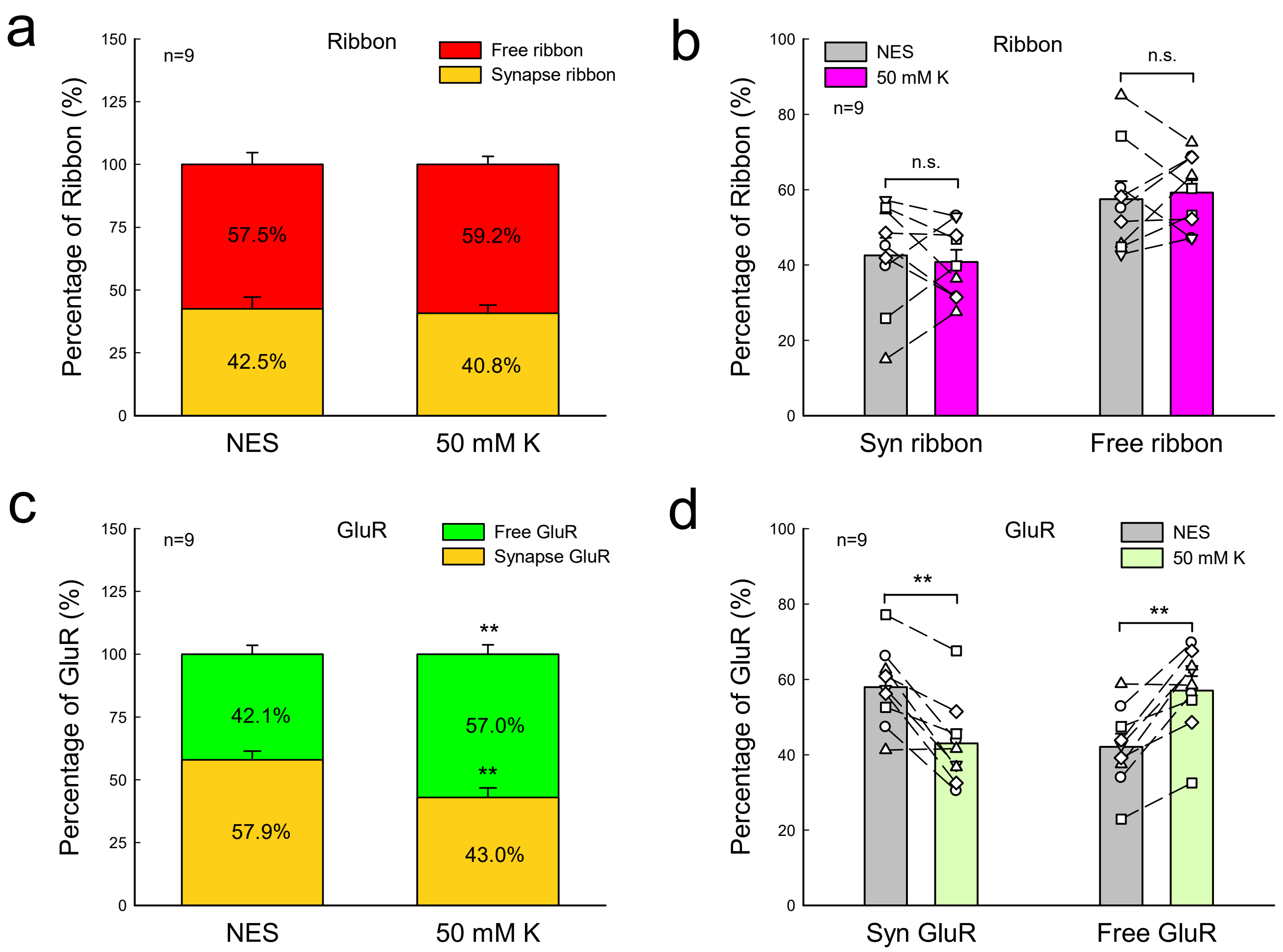

Supplementary Figure 1. Changes in synaptic ribbons, free ribbons, synaptic GluRs, and free GluRs after high-K^+ (50 mM) treatment.

The percentages of synaptic ribbons and free ribbons in the NES group and high-K^+ group were calculated by dividing total ribbon number in each group. The same calculation was also applied to GluRs.

a-b: Changes in synaptic and free ribbons after high-K^+ challenge. There are no significant changes in percentages of synaptic and free ribbons between the high-K^+ group and the NES control group, indicating that K^+ degenerated both synaptic and free ribbons. N is the animal number. P=0.68, paired t test, two-tail.

c-d: Changes in synaptic and free GluRs after high-K^+ challenge. The percentage of synaptic GluRs in the high-K^+ group is significantly reduced, indicating major loss of synaptic GluRs after high-K^+ challenge. **: P < 0.01, paired t test, two-tail.

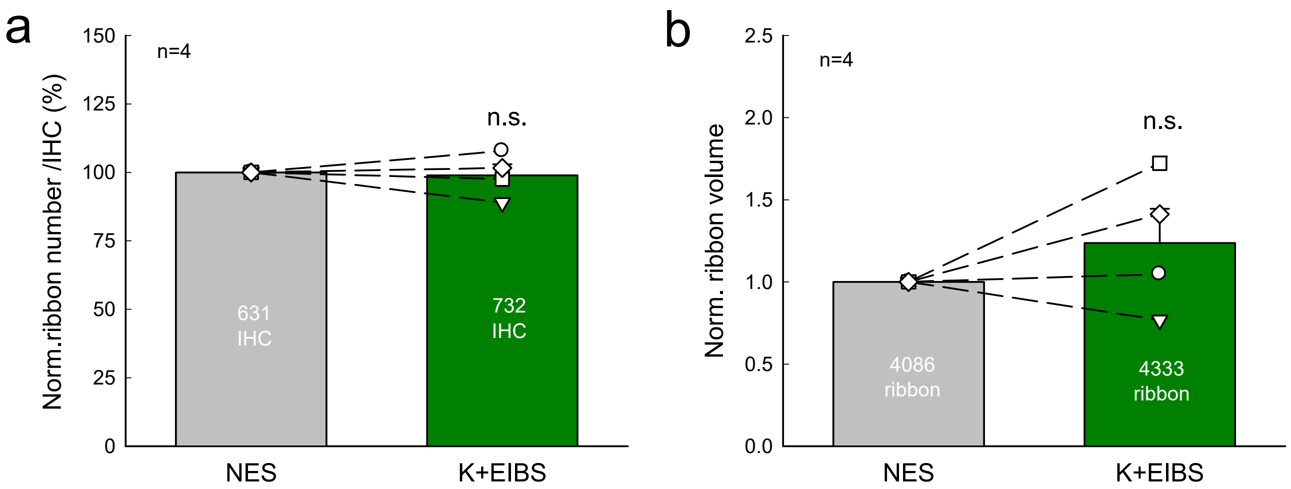

Supplementary Figure 2. Elimination of K^+-induced IHC ribbon degeneration and swelling by extracellular ionic blocking solution (EIBS) in the paired experiment with normal extracellular solution (NES) *vs* K^++EIBS .

One cochlea was incubated in EIBS with 50 mM K^+ (K^++EIBS) and another cochlea of the same mouse was incubated in the NES as control. The data were normalized to those in the control cochlea in the same mouse. N is the number of mice.

a: IHC ribbon numbers in the K^++EIBS group have no significant reduction for 50 mM K^+ challenge. There is no significant difference in IHC ribbon numbers between the K^++EIBS group and the NES control group. n.s.: no significance (P=0.52), paired t test, two-tail.

b: IHC ribbon volumes in the K^++EIBS group are not significantly increased for 50 mM K^+ challenge. There is no significant difference in ribbon volumes between the K^++EIBS group and the NES control group. n.s.: no significance (P=0.94), paired t test, two-tail.

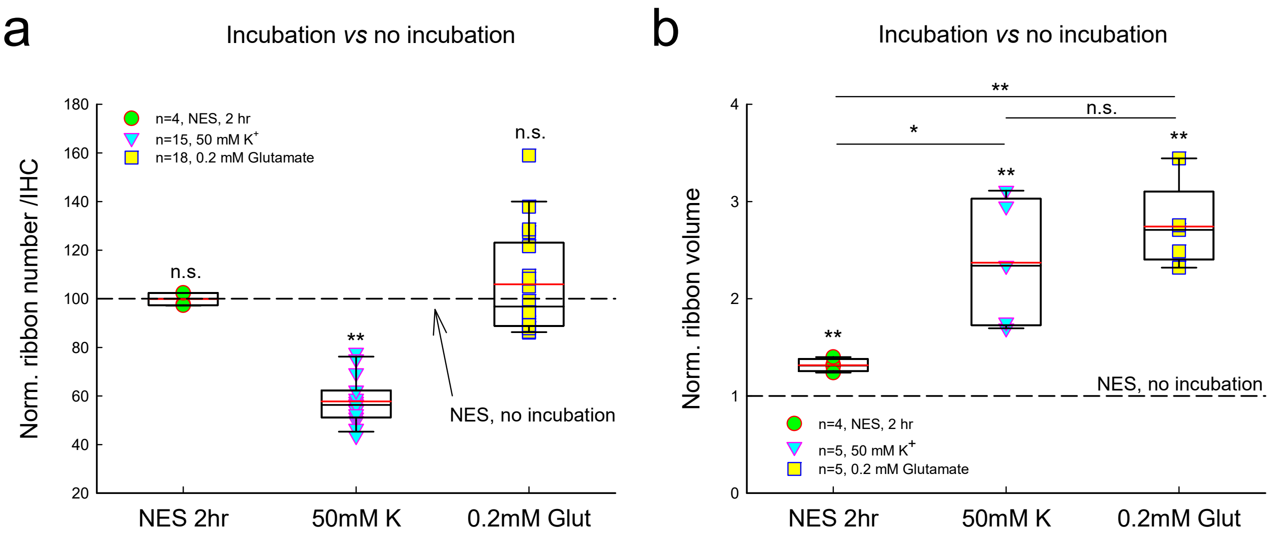

Supplementary Figure 3. Comparisons of effects of incubation procedure, high-K^+ (50 mM) challenge, and glutamate (0.2 mM) treatment on IHC ribbon number and volume.

The ribbon number per IHC and ribbon volume were normalized to those in the non-incubation control group, which are indicated by dashed lines in the figures. Red lines in the box represent the mean level. N is the number of mice.

a: The effect on ribbon numbers. In comparison with that in the non-incubation group (indicated by a dashed line), the normalized ribbon number per IHC after incubation with NES for 2 hr, 50 mM K^+ challenge, and 0.2 mM glutamate application was 99.9±1.40, 57.8±2.54, and 105.9±4.95%, respectively. Application of 50 mM K^+ caused significant reduction in IHC ribbons. However, incubation procedure and glutamate treatment had no significant effect on IHC ribbon degeneration. **: P<0.01, n.s.: no significance, P>0.05, one-way ANOVA with a Bonferroni correction.

b: The effect on ribbon volumes. In comparison with that in the non-incubation group (indicated by a dashed line), the IHC ribbon volumes in the incubation with NES for 2 hr, 50 mM K^+ challenge, and 0.2 mM glutamate treatment were significantly increased 1.32±0.03, 2.37±0.29, and 2.74±0.19 folds, respectively. The increases (2.37-2.74 folds) in 50 mM K^+ or 0.2 mM glutamate treatment were significantly larger than that (1.32 folds) in the incubation with NES for 2 hr. **: P<0.01; *: P<0.05; n.s.: no significance, P>=0.05, one-way ANOVA with a Bonferroni correction.
