# Supplementary material for: Excess extracellular K+ causes inner hair cell ribbon synapse degeneration
Source: Commun Biol. 2021 Jan 4;4:24. doi: 10.1038/s42003-020-01532-w (PMC7782724; doi:10.1038/s42003-020-01532-w)
Supplement: Supplementary file 2 — Description of Supplementary Files [file 42003_2020_1532_MOESM2_ESM.pdf]

Description of Additional Supplementary Files

File Name: Supplementary Data 1

Description: Source data for figures.
