# Supplementary material for: Excess extracellular K+ causes inner hair cell ribbon synapse degeneration
Source: Commun Biol. 2021 Jan 4;4:24. doi: 10.1038/s42003-020-01532-w (PMC7782724; doi:10.1038/s42003-020-01532-w)
Supplement: Supplementary file 4 — Reporting Summary [file 42003_2020_1532_MOESM4_ESM.pdf]

nature research

Corresponding author(s): Hong-Bo Zhao

Last updated by author(s): Sep 10, 2020

Reporting Summary

Nature Research wishes to improve the reproducibility of the work that we publish. This form provides structure for consistency and transparency in reporting. For further information on Nature Research policies, see our Editorial Policies and the Editorial Policy Checklist.

Statistics

For all statistical analyses, confirm that the following items are present in the figure legend, table legend, main text, or Methods section.

n/a	Confirmed	
☐	☒	The exact sample size (*n*) for each experimental group/condition, given as a discrete number and unit of measurement
☐	☒	A statement on whether measurements were taken from distinct samples or whether the same sample was measured repeatedly
☐	☒	The statistical test(s) used AND whether they are one- or two-sided<br>*Only common tests should be described solely by name; describe more complex techniques in the Methods section.*
☒	☐	A description of all covariates tested
☐	☒	A description of any assumptions or corrections, such as tests of normality and adjustment for multiple comparisons
☐	☒	A full description of the statistical parameters including central tendency (e.g. means) or other basic estimates (e.g. regression coefficient) AND variation (e.g. standard deviation) or associated estimates of uncertainty (e.g. confidence intervals)
☐	☒	For null hypothesis testing, the test statistic (e.g. *F*, *t*, *r*) with confidence intervals, effect sizes, degrees of freedom and *P* value noted<br>*Give P values as exact values whenever suitable.*
☒	☐	For Bayesian analysis, information on the choice of priors and Markov chain Monte Carlo settings
☒	☐	For hierarchical and complex designs, identification of the appropriate level for tests and full reporting of outcomes
☒	☐	Estimates of effect sizes (e.g. Cohen's *d*, Pearson's *r*), indicating how they were calculated

Our web collection on statistics for biologists contains articles on many of the points above.

Software and code

Policy information about availability of computer code

Data collection	Confocal images were captured by Nickon Element software (Nickon, version 4.5). ABR was recorded by BiosigRP software (Tucker-Davis Tech. version 4).
Data analysis	NIS Elements AR Analysis software (Nikon, version 4.5), Imaris (Bitplane Inc. version 9.3), Photoshop CC (Adobe), BiosigRP software (Tucker-Davis Tech. version 4), SigmaPlot (v10-13), Microsoft Excel (Microsoft office, 2016), and SPSS (SPSS Inc. V25).

For manuscripts utilizing custom algorithms or software that are central to the research but not yet described in published literature, software must be made available to editors and reviewers. We strongly encourage code deposition in a community repository (e.g. GitHub). See the Nature Research guidelines for submitting code & software for further information.

Data

Policy information about availability of data

All manuscripts must include a data availability statement. This statement should provide the following information, where applicable:

- Accession codes, unique identifiers, or web links for publicly available datasets
- A list of figures that have associated raw data
- A description of any restrictions on data availability

All source data underlying the graphs presented in the main figures are reported in the Source Data File. All data and materials produced by this study are available from the corresponding author upon request.

Field-specific reporting

Please select the one below that is the best fit for your research. If you are not sure, read the appropriate sections before making your selection.

- [x] Life sciences
- [ ] Behavioural & social sciences
- [ ] Ecological, evolutionary & environmental sciences

For a reference copy of the document with all sections, see nature.com/documents/nr-reporting-summary-flat.pdf

Life sciences study design

All studies must disclose on these points even when the disclosure is negative.

Sample size	Sample size was determined based on the research term's prior experience.
Data exclusions	No data were excluded from the analyses. However, since ribbon fluorescent labeling had apparent diffusion after long-term storage, one mouse was excluded from ribbon volume measurement in Fig. 4B for high-K and Co experiences, respectively.
Replication	Each in vitro experiment was repeated at least 4 times to verify the reproducibility. For in vivo noise exposure experiment, only one or two mice were exposed at each time. Totally, 3 times were repeated and 5 mice were exposed.
Randomization	Mice with either genders were randomly selected to be noise-exposure group and control group. Right and left ears were also randomly selected for high-K challenge or control.
Blinding	There was no blinding during data collection since most experiments were did by one scientist at a time. However, the blinding was applied in some cases during data analyses when different scientists processed data.

Reporting for specific materials, systems and methods

We require information from authors about some types of materials, experimental systems and methods used in many studies. Here, indicate whether each material, system or method listed is relevant to your study. If you are not sure if a list item applies to your research, read the appropriate section before selecting a response.

Materials & experimental systems

n/a	Involved in the study	
☐	☒	Antibodies
☒	☐	Eukaryotic cell lines
☒	☐	Palaeontology and archaeology
☐	☒	Animals and other organisms
☒	☐	Human research participants
☒	☐	Clinical data
☒	☐	Dual use research of concern

Methods

n/a	Involved in the study	
☒	☐	ChIP-seq
☒	☐	Flow cytometry
☒	☐	MRI-based neuroimaging

Antibodies

Antibodies used	mouse anti-CtBP2 IgG1 (1:200-500, #612044, BD Bioscience), mouse anti-GluR2 IgG2a (1:500-1000, #MAB397, Millipore Corp.), goat anti-mouse IgG2a Alexa Fluor 488 (1:500, A-21131, Thermo Fisher Sci), goat anti-mouse IgG1 Alexa Fluor 568 (1:500, A-21124, hermo Fisher Sci).
Validation	The specificity of these antibodies were verified by previous reports, such as Kujawa and Liberman (J of Neuroscience 29: 14077-14085, 2009) and Shrestha et al. (Cell 174: 1229-1246, 2018)

Animals and other organisms

Policy information about studies involving animals; ARRIVE guidelines recommended for reporting animal research

Laboratory animals	Both genders, adult (8-12 weeks old) CBA/CaJ mice (Stock No: 000654, Jackson Lab, USA) were used.
Wild animals	N/A
Field-collected samples	N/A
Ethics oversight	All procedures and experiments of use of animals were approved by the University of Kentucky's Animal Care & Use Committee (UK: 00902M2005) and conformed to the standards of the NIH Guidelines for the Care and Use of Laboratory Animals.

Note that full information on the approval of the study protocol must also be provided in the manuscript.
